# Lagged Associations of Metropolitan Statistical Area- and State-Level Income Inequality with Cognitive Function: The Health and Retirement Study

**DOI:** 10.1371/journal.pone.0157327

**Published:** 2016-06-22

**Authors:** Daniel Kim, Beth Ann Griffin, Mohammed Kabeto, José Escarce, Kenneth M. Langa, Regina A. Shih

**Affiliations:** 1 Department of Health Sciences, Bouvé College of Health Sciences, Northeastern University, Boston, Massachusetts, United States of America; 2 RAND Corporation, Santa Monica, California, United States of America; 3 University of Michigan Health System, Ann Arbor, Michigan, United States of America; 4 Department of Social and Behavioral Sciences, Harvard T.H. Chan School of Public Health, Boston, Massachusetts, United States of America; 5 Department of Social and Behavioral Sciences, EHESP School of Public Health, Sorbonne Paris Cité, Paris Descartes University, Paris, France; 6 UCLA David Geffen School of Medicine, Los Angeles, California, United States of America; University of California San Francisco, UNITED STATES

## Abstract

**Purpose:**

Much variation in individual-level cognitive function in late life remains unexplained, with little exploration of area-level/contextual factors to date. Income inequality is a contextual factor that may plausibly influence cognitive function.

**Methods:**

In a nationally-representative cohort of older Americans from the Health and Retirement Study, we examined state- and metropolitan statistical area (MSA)-level income inequality as predictors of individual-level cognitive function measured by the 27-point Telephone Interview for Cognitive Status (TICS-m) scale. We modeled latency periods of 8–20 years, and controlled for state-/metropolitan statistical area (MSA)-level and individual-level factors.

**Results:**

Higher MSA-level income inequality predicted lower cognitive function 16–18 years later. Using a 16-year lag, living in a MSA in the highest income inequality quartile predicted a 0.9-point lower TICS-m score (β = -0.86; 95% CI = -1.41, -0.31), roughly equivalent to the magnitude associated with five years of aging. We observed no associations for state-level income inequality. The findings were robust to sensitivity analyses using propensity score methods.

**Conclusions:**

Among older Americans, MSA-level income inequality appears to influence cognitive function nearly two decades later. Policies reducing income inequality levels within cities may help address the growing burden of declining cognitive function among older populations within the United States.

## Introduction

Epidemiological studies suggest that cognitive impairment and decline are linked to individual-level characteristics including age [1], race/ethnicity [2, 3], education/socioeconomic status [4–7], vascular conditions [8, 9], and social integration/support [10]. Yet much of the variation in cognitive function remains unexplained, and could partly be attributed to area-level/contextual factors. To date, exploration of contextual factors as predictors of cognitive function has been limited to neighborhood socioeconomic status (SES) [11–13].

Income inequality, the degree of unequal distribution of income within populations, is an area-level/contextual factor hypothesized to adversely affect health [14–16]. Proposed mechanisms include individuals’ feelings of relative deprivation; erosion of collective social cohesion and trust; and disinvestments in public goods such as education and health care, as interests of the rich diverge from those of the poor [14, 15]. Plausibly, some of these mechanisms may relate to cognitive function, such as through influencing behavioral and psychosocial factors including levels of social support [10], depressive symptoms [17], or one’s diet [18], in turn influencing cognition.

While the effects of income inequality on health have been extensively studied, the literature is mixed. In a meta-analysis of multilevel cohort studies, higher within-country income inequality predicted a 1.08 times higher risk of individual-level mortality [19]. Other review studies suggest stronger health effects of income inequality at larger geographical scales e.g., states [vs. metropolitan statistical area (MSA)/counties] within the United States (U.S.) [20] or countries [15]. This may signify policy-related mechanisms, such as economic disparities contributing to patterns of spending by state legislatures on public goods including education and welfare [20, 21]. While prior studies have examined various health endpoints such as self-reported health and mortality, no study has yet examined the associations of income inequality with cognitive function, a more proximal outcome than mortality. Furthermore, no individual study has simultaneously estimated health associations of income inequality at the MSA and state levels.

The effects of income inequality on cognitive function plausibly vary by the time period separating the measures of income inequality and cognitive function (i.e., the lag period). For example, as with many chronic diseases, the pathological process of cognitive impairment likely begins years prior to being expressed clinically in late adulthood (e.g., in the late 60s or early 70s). The "cognitive reserve" hypothesis posits that exposure to environmental factors that provide more cognitive enrichment such as higher educational attainment may help build a higher cognitive reserve of nervous system functioning and confer a resilience to neuropathological insults (e.g., vascular damage, Alzheimer's protein build-up, inflammation), such that a higher neuropathological threshold must be reached before impairment of cognitive function manifests clinically [22, 23]. Studies have consistently linked a higher level of education to a lower risk of Alzheimer's Disease [24,25]. This may reflect the extent of early cognitive stimulation of the brain, which in turn may influence cognitive function [26]. In addition, cognitive reserve and cognitive impairment may be determined by other indicators of socioeconomic status such as income and occupation [27, 28], or by lifestyle behaviors and leisure activities [29,30]. Some evidence suggests that the effects of state-level income inequality on individual self-rated health are strongest after a latency period of roughly 16 years, with weaker effects measured 4–12 years prior to self-rated health [31]. Similarly, the effects of income inequality on cognitive function likely manifest after several years rather than instantaneously, though have yet to be empirically tested in lagged effect models. We hypothesized that the effects would be most sensitive with temporally-specific lag periods, as has been observed for other exposure-outcome relationships. For example, air pollution exposures have been shown to exhibit temporally-specific lag periods with cardiovascular outcomes [32].

Using nationally representative data on older Americans from the Health and Retirement Study (HRS), we examined the lagged associations of income inequality measured at both the MSA and state levels with individual-level cognitive function, controlling for state-/MSA-level and individual-level factors.

## Materials and Methods

### Study population

The Health and Retirement Study (HRS) is a nationally-representative prospective cohort study of U.S. adults born in 1947 or earlier, designed to investigate health, social, and economic outcomes in aging Americans. The original HRS study cohort that served as our study population consisted of 10,620 participants who were members of households with at least one person born during the period 1931–1941 i.e., aged 51–61 years in 1992. Addresses were geocoded to 1990 Census boundaries based on the MSA/state of residence in 1992; 134 (1.3%) participants could not be geocoded. Of the remaining cohort, 2,573 (24.2%) participants lived in a non-MSA at baseline and were excluded from the MSA-level analyses (but included in state-level analyses). An additional 392 (3.7%) participants were missing baseline Gini coefficient data for the MSA-level analyses and omitted from these analyses. For the state-level analyses, no additional participants had missing Gini coefficient data; 24 residents of the District of Columbia were excluded. Finally, 1,353 (12.7%) participants and 1,841 (17.3%) participants from the MSA- and state-level analyses, respectively, could not be interviewed because they were too impaired to complete the cognitive assessments, resulting in a proxy respondent being interviewed. These individuals were omitted because the cognitive outcome used for primary respondents was not administered to proxy respondents; the exclusion of these participants from our analyses is unfortunate. Study participants with follow-up assessments were retained in the study sample regardless of subsequent moves to a different MSA or state at follow-up than at baseline in 1992. The final baseline sample eligible for the MSA-level analyses was comprised of 5,455 adults in 1992; the final sample eligible for the state-level analyses consisted of 7,568 adults in 1992.

### Outcome variable

We used a modified version of the Telephone Interview for Cognitive Status (TICS-m), a validated measure of global cognitive function, administered at each wave between 1998 and 2010 [33, 34]. The main HRS cognitive test is a 27-point scale that includes immediate and delayed word recall of a list of 10 nouns; working memory is assessed using the Serial 7’s subtraction test from 100; and knowledge, language, and orientation are assessed by counting backward for 10 continuous numbers beginning with 20 as quickly as possible. The TICS-m score was calculated as the sum of the component scores (range = 0–27), and modeled as a continuous variable (higher scores signifying higher cognitive function).

### Predictor variable

Income inequality was modeled as a predictor of cognitive function with the Gini coefficient. Theoretical values for the Gini coefficient range from 0, reflecting perfect equality, to 1, corresponding to perfect inequality [15]. We examined the Gini coefficient, derived from household income data from the 1990 US Census, at two levels of aggregation: the state level and the MSA level. The 1990 value of the Gini coefficient was assigned to each study participant’s geocoded MSA/state of residence, based on addresses reported in 1992. The Gini coefficient was modeled as quartile categories, using all U.S. MSAs/states with available Gini coefficient data (304 MSAs/50 states plus the District of Columbia—see S1 and S2 Tables), to explore potential non-linear effects on cognitive function.

### Covariates

All linear regression models were adjusted for multiple factors at the individual and area levels that could confound the relationship between income inequality and cognitive function. These factors consisted of 1992 baseline age (range: 51–61 years; modeled as a continuous variable), gender, race/ethnicity (non-Hispanic White, non-Hispanic Black, Hispanic, Other), education (<high school, high school, greater than high school education), and net wealth (≤$41,600, $41,601–138,000, $138,001–343,000, >$343,000). Models also controlled for baseline self-reported medical diagnoses of diabetes, hypertension, heart disease, and stroke, and measures of body mass index. The earliest measure of TICS-m in 1995/1996 was further included in all models as a proxy for baseline cognitive function. We also controlled for the 1990 Census-derived percentage Black and median household income measured at the same geographic level as the respective Gini coefficient.

### Statistical analysis

We calculated descriptive statistics for all individual- and MSA/state-level characteristics of the baseline analytic samples. We then plotted the marginal means of the TICS-m scores (i.e., the mean TICS-m scores at the mean values of the covariates) for those residing within each MSA/state Gini coefficient quartile across biennial years 1998 through 2010.

Using multivariate linear regression, we next incorporated varying lag periods between the baseline value of the Gini coefficient and cognitive function measures (in 2-year increments, from 8 to 20 years). The lag period is the number of years between the 1990 income inequality exposure (assuming that the study participant lived in the same MSA/state in 1990 as at the baseline survey in 1992) and the TICS-m score year. In separate models, the TICS-m score measured in year *t* (where *t =* 1998, 2000, 2002, 2004, 2006, 2008 and 2010) was regressed as a continuous variable on the quartile of income inequality of MSA/state residence and the individual and MSA-/state-level covariates. The quartile of lowest MSA-/state-level income inequality served as reference group. All results were weighted using sampling weights at baseline in 1992 to account for the complex multistage survey design, including stratification, clustering, and additional post-stratification.

All analyses incorporated attrition weights to adjust for non-response bias (unweighted analysis results are reported in S3 and S4 Tables). Attrition weights were estimated using the TWANG package in SAS [35], which uses a non-parametric, machine learning estimation method to estimate attrition weights with better balancing properties than ordinary logistic regression models. Linear tests for trend were performed by modeling the Gini coefficient quartiles as a 4-level ordinal variable. We assessed the main effects point estimates and presence of linear trends using a 1% significance level, to account for multiple testing across TICS-m score models.

For each TICS-m score model, we tested for effect modification of income inequality effects by individual-level race/ethnicity, wealth, gender, and MSA/non-MSA residence (the latter in state-level analyses only). Specifically, we included and tested in each model the significance of the interaction terms for the cross-product of each factor with the income inequality quartiles. We used joint F-tests to assess the statistical significance of each set of interactions, applying a 1% significance level in light of multiple testing.

Finally, we performed sensitivity analyses using propensity score methods to reduce potential bias due to non-random selection of individuals into quartiles of income inequality (i.e., MSAs and states) by observable factors. Using TWANG [36], Generalized Boosted Models were estimated to generate the conditional probability (propensity score) of being in an income inequality quartile given individual-level covariates—the same individual-level covariates included in the multiple linear regression models. These estimated propensity scores were then used to compute weights to enable balance on all of these covariates (and their associated interactions and higher-order terms) across income inequality quartiles. To take into account migration into a different MSA over the study period, we further performed sensitivity analyses in which we repeated the MSA analyses after excluding all individuals who moved from the MSA in which the study participant resided at the baseline survey in 1992 to a different MSA in any of the HRS biennial years 1994 through 2010. In addition, we performed sensitivity analyses that restricted to the same sample of participants assessed for cognitive function at every wave from 1998 to 2010.

HRS participants provided verbal informed consent. All respondents were read a confidentiality statement when first contacted, and gave oral or implied consent by agreeing to do the interview. Verbal consent was obtained because most interviews were conducted by telephone. HRS staff at the University of Michigan recorded consent in writing. The Institutional Review Board (IRB) at the University of Michigan approved the methods, consent procedure, and data collection in the HRS. The IRB at the RAND (Research ANd Development) Corporation also approved analysis of these data.

## Results

### Descriptive characteristics

At baseline in 1992, participants from the original HRS cohort in the MSA-level analytic sample ranged in age from 51–61 years; 55.2% were female, 80.2% were White, and 42.9% had attained high school education (Table 1). The mean baseline TICS-m score was 17.0 (range: 0–27). The median 1990 state-level Gini coefficient was 0.44 (range: 0.41–0.48). Study participants lived in 76 MSAs, with a median MSA-level Gini coefficient of 0.43 (range: 0.37–0.49). With baseline samples as reference, follow-up rates in the MSA-level analyses ranged from 61.6% to 88.8% between 1998 and 2010; in the state-level analyses, follow-up rates ranged from 62.0% to 89.3%.

**Table 1:** Descriptive characteristics of MSA- and state-level analytic samples at baseline (1992), Health and Retirement Study.

**MSA- or State-Level Characteristics**	**MSA-Level Analytic Sample (n = 5,455)**		**State-Level Analytic Sample (n = 7,568)**
1990 MSA-Level Gini Coefficient Quartiles		1990 State-Level Gini Coefficient Quartiles	
Q1 (Ref): 0.362–0.409	552 (11.4%)	Q1 (Ref): 0.390–0.414	663 (8.8%)
Q2: 0.409–0.426	1,607 (29.8%)	Q2: 0.414–0.428	996 (13.2%)
Q3: 0.426–0.444	1,471 (26.7%)	Q3: 0.428–0.445	2,990 (39.5%)
Q4: >0.444	1,825 (32.0%)	Q4: >0.445	2,919 (38.6%)
MSA-Level % Black (SE)	13.1 (0.54)	State-Level % Black (SE)	11.7 (0.47)
MSA-Level Median Household Income (SE)	$32,146 (526)	State-Level Median Household Income (SE)	$30,347 (351)
**Individual-Level Characteristics**			
Mean TICS-m Score (SE) in 1995/1996	17.0 (0.13)		16.9 (0.10)
Age (years)			
51–55	2,693 (49.4%)		3,721 (49.2%)
56–61	2,762 (50.6%)		3,847 (50.8%)
Gender			
Male	2,371 (44.8%)		3,319 (43.9%)
Female	3,084 (55.2%)		4,249 (56.1%)
Education (years)			
<12	1,404 (21.7%)		2,057 (27.2%)
12	1,888 (35.4%)		2,727 (36.0%)
13+	2,163 (42.9%)		2,784 (36.8%)
Race/Ethnicity			
White	3,794 (80.2%)		5,603 (74.0%)
Black	1,059 (11.4%)		1,230 (16.3%)
Hispanic	523 (6.6%)		632 (8.4%)
Other	79 (1.7%)		103 (1.4%)
Net Wealth			
≤ $41,600	1,740 (27.5%)		2,373 (31.4%)
$41,601 –$138,000	1,764 (31.7%)		2,540 (33.6%)
$138,001 –$343,000	1,272 (25.9%)		1,710 (22.6%)
>$343,000	679 (14.8%)		945 (12.5%)
Health Condition			
Hypertension	2,129 (36.9%)		2,974 (39.2%)
Stroke	145 (2.3%)		204 (2.7%)
Heart Disease	668 (12.0%)		941 (12.4%)
Diabetes	567 (9.4%)		778 (10.3%)
Body Mass Index (kg/m^2^)			
<19	88 (1.5%)		124 (1.6%)
19–25	1,903 (37.0%)		2,624 (34.6%)
25.1–30	2,214 (40.5%)		3,068 (40.4%)
>30	1,250 (21.0%)		1,769 (23.3%)

For continuous variables, mean or median values (with standard errors in parentheses) are displayed. For categorical variables, the frequencies (with percentage of the sample in parentheses) are shown in each category.

Quartile 1 (Q1) for the Gini coefficient (reference category) corresponds to the quartile of US MSAs/states with the lowest Gini coefficient (lowest level of income inequality), based on 304 MSAs and all 50 states with available Gini coefficient data.

**Fig 1:**
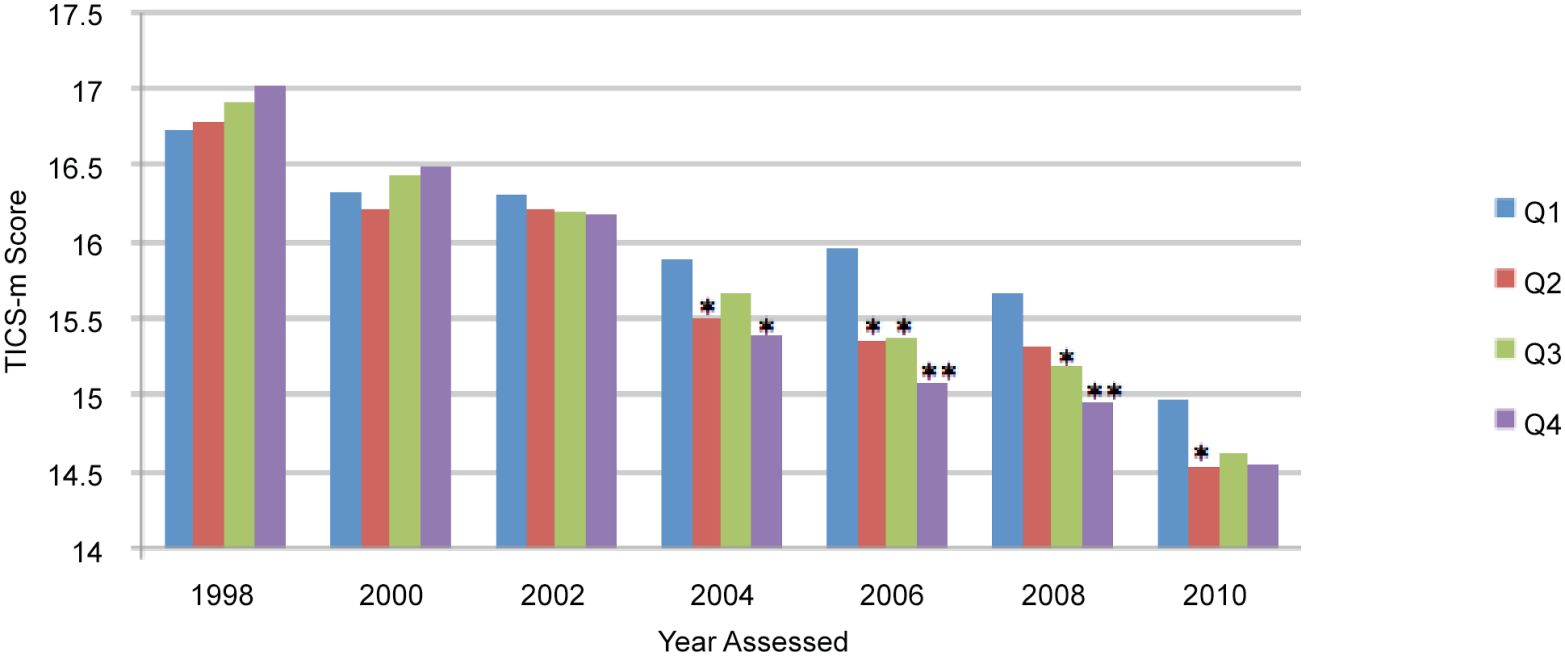
Marginal TICS-m scores between 1998 and 2010 corresponding to residence in quartiles of 1990 MSA-level income inequality, Health and Retirement Study. Adjusted for baseline age, gender, race/ethnicity, education, net wealth, self-reported medical diagnoses of diabetes, hypertension, heart disease, and stroke, body mass index, and the 1995/1996 TICS score; and the percentage Black and median household income at the MSA level. Quartile 1 (Q1) for the Gini coefficient (reference category) corresponds to the quartile of US MSAs with the lowest Gini coefficients (lowest level of income inequality), based on 304 MSAs with available Gini coefficient data. * P≤.05; ** P≤.01 for comparison of marginal TICS-m scores for those living in the respective higher income inequality quartile (Q2, Q3, or Q4) to scores for those in Q1.

Between 1998 and 2010, with each higher income inequality quartile, we observed a gradual decrease in the means of the TICS-m scores (Fig 1). For 1998, 2000, and 2002, there were no significant differences in the marginal mean TICS-m scores between those living in MSAs in any of the three higher income inequality quartiles (Q2-Q4) relative to those in the lowest income inequality quartile (Q1). In contrast, in 2004, 2006, and 2008, the marginal mean TICS-m scores were significantly lower for those living in MSAs belonging to Q4 relative to those in Q1; there was a monotonic dose-response relationship in 2006 and 2008 (Fig 1).

### Lagged associations between MSA-level income inequality and cognitive function

Findings from the models that examined MSA-level income inequality are shown in Table 2. For TICS-m outcomes with lag periods of ≥12 years, the respective associations between income inequality and cognitive function were consistently inverse. In the models using 16- and 18-year lag periods, there was evidence of significant inverse associations in the quartiles corresponding to the highest (Q4) and second (Q3) highest levels of income inequality. For example, living in a MSA in the highest (vs. lowest) quartile of income inequality in 1990 predicted a 0.86-point and 0.70-point lower cognitive score 16 and 18 years later, respectively. For each of these outcome years, we observed a significant linear trend across quartiles (Table 2). Moreover, the point estimates for Q4 (vs. Q1) for income inequality corresponded to 12, 14, and 20-year lagged associations with magnitudes of -0.13, -0.48, and -0.39, respectively, consistent with a temporal pattern.

**Table 2:** Non-response weighted multivariate linear regression coefficient estimates with varying lag periods between the 1990 U.S. MSA-level Gini coefficient and TICS-m score (1998–2010), Health and Retirement Study.

		HRS Cohort (n = 5,455 in 1992)	
		β	95% CI
**1998 TICS, 8-year lag**	**n = 4,842 (88.8%)**		
	**Quartile of 1990 Gini**		
	Q2	0.05	-0.39, 0.49
	Q3	0.17	-0.34, 0.69
	Q4	0.30	-0.15, 0.74
	*P for trend = 0*.*10*		
**2000 TICS, 10-year lag**	**n = 4,507 (82.6%)**		
	**Quartile of 1990 Gini**		
	Q2	-0.12	-0.49, 0.26
	Q3	0.11	-0.31, 0.53
	Q4	0.16	-0.25, 0.56
	*P for trend = 0*.*11*		
**2002 TICS, 12-year lag**	**n = 4,272 (78.3%)**		
	**Quartile of 1990 Gini**		
	Q2	-0.08	-0.49,0.33
	Q3	-0.11	-0.59,0.37
	Q4	-0.13	-0.58,0.33
	*P for trend = 0*.*59*		
**2004 TICS, 14-year lag**	**n = 4,077 (74.7%)**		
	**Quartile of 1990 Gini**		
	Q2	-0.36	-0.75,0.02
	Q3	-0.23	-0.66,0.19
	Q4	-0.48*	-0.88,-0.07
	*P for trend = 0*.*08*		
**2006 TICS, 16-year lag**	**n = 3,914 (71.7%)**		
	**Quartile of 1990 Gini**		
	Q2	-0.59*	-1.11,-0.06
	Q3	-0.59*	-1.14,-0.04
	Q4	-0.86**	-1.41,-0.31
	*P for trend = 0*.01**		
**2008 TICS, 18-year lag**	**n = 3,683 (67.5%)**		
	**Quartile of 1990 Gini**		
	Q2	-0.32	-0.81,0.17
	Q3	-0.49*	-0.92,-0.05
	Q4	-0.70**	-1.18,-0.23
	*P for trend = 0*.01**		
**2010 TICS, 20-year lag**	**n = 3,361 (61.6%)**		
	**Quartile of 1990 Gini**		
	Q2	-0.40*	-0.79,-0.01
	Q3	-0.33	-0.74,0.08
	Q4	-0.39	-0.94,0.16
	*P for trend = 0*.*42*		

Note. β = coefficient estimate; CI = confidence interval. Q = quartile of 1990 MSA Gini coefficient.

All models are adjusted for baseline age, gender, race/ethnicity, education, net wealth, self-reported medical diagnoses of diabetes, hypertension, heart disease, and stroke, body mass index, and the 1995/1996 TICS-m score; and the percentage Black and median household income at the MSA level. Quartile 1 (Q1) for the Gini coefficient (reference category) corresponds to the quartile of US MSAs with the lowest Gini coefficient (lowest level of income inequality), based on 304 MSAs with available Gini coefficient data in 1990. Sample sizes are shown for each outcome year (the percentage of the baseline sample is shown in parentheses).

* P≤.05;

** P≤.01.

### Lagged associations between state-level income inequality and cognitive function

Findings based on the models with the state-level Gini coefficient are displayed in Table 3. Higher degrees of state-level income inequality in 1990 predicted higher cognitive scores ≥14 years later, but all associations were statistically non-significant. No significant linear trends were observed in any model (Table 3).

**Table 3:** Non-response weighted multivariate linear regression coefficient estimates with varying lag periods between the 1990 U.S. state-level Gini coefficient and TICS-m score (1998–2010), Health and Retirement Study.

		HRS Cohort (n = 7,568 in 1992)	
		β	95% CI
**1998 TICS, 8-year lag**	**n = 6,761 (89.3%)**		
	**Quartile of 1990 Gini**		
	Q2	-0.34	-0.81, 0.12
	Q3	0.04	-0.33, 0.40
	Q4	0.02	-0.42, 0.46
	*P for trend = 0*.*34*		
**2000 TICS, 10-year lag**	**n = 6,265 (82.8%)**		
	**Quartile of 1990 Gini**		
	Q2	-0.26	-0.75, 0.23
	Q3	-0.13	-0.60, 0.34
	Q4	-0.09	-0.64, 0.45
	*P for trend = 0*.*99*		
**2002 TICS, 12-year lag**	**n = 5,919 (78.2%)**		
	**Quartile of 1990 Gini**		
	Q2	-0.09	-0.49,0.32
	Q3	-0.14	-0.48,0.20
	Q4	0.01	-0.43,0.46
	*P for trend = 0*.*94*		
**2004 TICS, 14-year lag**	**n = 5,683 (75.1%)**		
	**Quartile of 1990 Gini**		
	Q2	0.05	-0.41,0.52
	Q3	0.04	-0.25,0.33
	Q4	0.12	-0.20,0.43
	*P for trend = 0*.*57*		
**2006 TICS, 16-year lag**	**n = 5,476 (72.3%)**		
	**Quartile of 1990 Gini**		
	Q2	0.31	-0.13,0.75
	Q3	0.28	-0.08,0.65
	Q4	0.08	-0.29,0.46
	*P for trend = 0*.*97*		
**2008 TICS, 18-year lag**	**n = 5,162 (68.2%)**		
	**Quartile of 1990 Gini**		
	Q2	0.20	-0.36,0.75
	Q3	0.08	-0.19,0.36
	Q4	-0.06	-0.49,0.38
	*P for trend = 0*.*55*		
**2010 TICS, 20-year lag**	**n = 4,694 (62.0%)**		
	**Quartile of 1990 Gini**		
	Q2	0.16	-0.32,0.64
	Q3	0.20	-0.17,0.57
	Q4	0.07	-0.32,0.45
	*P for trend = 0*.*84*		

Note. β = coefficient estimate; CI = confidence interval. Q = quartile of 1990 state Gini coefficient.

All models are adjusted for baseline age, gender, race/ethnicity, education, net wealth, self-reported medical diagnoses of diabetes, hypertension, heart disease, and stroke, body mass index, and the 1995/1996 TICS-m score; and the percentage Black and median household income at the state level. Quartile 1 (Q1) or the Gini coefficient (reference category) corresponds to the quartile of US states with the lowest Gini coefficients (lowest level of income inequality), based on all 50 states in 1990. Sample sizes are shown for each outcome year and cohort (the percentage of the baseline sample is shown in parentheses).

### Effect modification by individual-level factors and MSA/non-MSA residence

We found no consistent and significant interactions between gender, race/ethnicity, and net wealth with MSA-level income inequality in their estimated effects on cognitive function (data not shown). Furthermore, we did not identify any interactions between gender, net wealth, or living in a MSA (vs. non-MSA) area with state-level income inequality.

### Sensitivity analyses

In sensitivity analyses using propensity scores, we obtained qualitatively similar results to those shown in Tables 2 and 3, with no differences in statistical significance at a 1% level (data not shown). In sensitivity analyses restricted to non-movers (86.1% of the full analytic sample at baseline) over the 1992–2010 time period, we found relatively comparable corresponding results in terms of direction, magnitude, and statistical significance to those obtained for the full samples. We likewise observed qualitatively similar results when we restricted the sample to those individuals cognitively assessed at every wave from 1998 to 2010, with no differences in statistical significance at the 1% level (see S5 and S6 Tables).

## Discussion

In a large, nationally representative, prospective cohort of older Americans, we found independent, graded associations of higher MSA-level income inequality with lower cognitive function measured at ages 67–79, using lag periods of 16 to 18 years. Living in a MSA in the quartile of highest income inequality was independently associated with a 0.9-point lower level of cognitive function 16 years later. Based on the marginal TICS-m scores we observed across waves, this effect size roughly corresponds to the magnitude associated with five years of aging.

Our study is the first to explore the relation between income inequality and cognitive function; past studies of contextual determinants of cognitive function have been confined to neighborhood SES. Furthermore, these studies have either been cross-sectional in design [11–13] or not nationally representative [37], thereby limiting the generalizability of findings.

Although prior studies of income inequality on mortality and self-rated health have identified stronger effects at the state than MSA or county levels, our findings support associations of income inequality at the MSA but not state level; in analyses of state-level income inequality, we observed no differences in income inequality associations according to residence in MSA vs. non-MSA areas. Given this evidence compatible with a geographic specificity of income inequality effects at the MSA level (vs. the state level), the MSA-level income inequality associations could reflect pathways acting at more local levels. If feelings of relative deprivation are primarily experienced through comparisons with others locally within the same metropolitan area (e.g., those encountered in everyday life) rather than comparisons with others across one’s state, the negative consequences of relative deprivation on cognition (e.g., mediated by depressive symptoms) would be expected to be more salient with higher levels of income inequality at the metropolitan level. Past evidence has linked perceptions of local relative deprivation to mental well-being [38].

Modeling income inequality measured several years prior to cognitive function assessment, we identified a temporal relation in our results. Furthermore, by modeling a range of lag periods, we determined the lag periods with the strongest associations on cognitive function in late life. Additional strengths of our analysis include the use of nationally-representative samples of older adults; re-weighting at the analysis stage for non-response to reduce selection bias; control for multiple area- and individual-level factors including co-morbid conditions and baseline cognitive function to limit residual confounding; exploration of associations at both the MSA and state levels; and sensitivity analyses using propensity score weights to further minimize selection bias.

Our significant associations of income inequality with cognitive function corresponded to lag periods of 16 and 18 years. These lagged associations for income inequality measured in mid-life are in keeping with previous work exploring lag periods between area-level income inequality and self-rated health, and support the manifestation of effects of income inequality on cognitive function after more than a decade. This temporal association plausibly reflects the average time that it takes for income inequality to adversely affect individual cognitive function, for example mediated by feelings of relative deprivation and psychosocial factors such as depression, or mediated by the erosion of collective social capital and trust, which in turn may influence behavioral and psychosocial determinants of cognitive function.

Our study had several limitations. First, income inequality assigned to study participants was based on the MSA/state of residence at baseline in 1992. Some study participants may have moved into other MSAs/states when they were cognitively assessed between 1998 and 2010. Our sensitivity analysis restricted to non-movers further indicated the robustness of our findings to migration to a different MSA over the study period. Second, both for the lack of observed associations for state-level income inequality and presence of associations for MSA-level income inequality, we cannot rule out residual confounding by income inequality or area-level SES (e.g., median household income) at smaller geographical scales (e.g., neighborhoods). Third, although we accounted for non-response through survey weights and conducted sensitivity analyses using propensity score methods (the results of which suggested robustness to self-selection of individuals into quartiles of MSA/states) and by restricting to the sample of individuals who were cognitively assessed in every wave from 1998 to 2010 (the results of which suggested robustness to whether the same individuals were assessed at all waves), none of these approaches eliminates selection bias due to unobservable factors. Selection bias could partly explain some of the observed null findings, if attrition/omission due to disability (e.g., in the case of proxy respondents) or deaths removed those most susceptible to cognitive decline from the analytic sample. If the remaining cohort members were characterized by higher cognitive reserve capacities, they may not have been as susceptible to neuropathological insults of income inequality. However, the significant findings of income inequality at the MSA level (with a 71% overlap of participants in the MSA- and state-level analysis samples) coupled with the apparent absence of effect modification by MSA/non-MSA residence in the state-level analyses do not favor selection bias due to attrition as a major explanation of the null state-level findings, and lend support to the presence of true effects of income inequality at the metropolitan level. Finally, while we controlled for multiple baseline co-morbid conditions to reduce confounding, we did not adjust for time-varying health events between the measures of income inequality and cognitive function, which could have led to residual confounding.

In summary, this study offers novel evidence on the associations of MSA-level income inequality with cognitive function nearly two decades later in a nationally-representative sample of older Americans. These findings may aid policymakers in identifying vulnerable populations at risk of long-term adverse effects on cognitive function from mid-life exposure to high levels of income inequality. By investigating possible mediating pathways, we may better elucidate how income inequality within cities shapes cognitive function. Policies that reduce levels of income inequality at the metropolitan level may be potential levers to address the growing public health burden of declining cognitive function among older populations within the United States.

## Supporting Information

S1 Table 1990 Gini Coefficient and Income Inequality Quartiles for U.S. MSAs. (XLSX)

S2 Table 1990 Gini Coefficient and Income Inequality Quartiles for U.S. States and D.C. (XLSX)

S3 Table Unweighted multivariate linear regression coefficient estimates with varying lag periods between the 1990 U.S. MSA-level Gini coefficient and TICS-m score (1998–2010), Health and Retirement Study. (DOCX)

S4 Table Unweighted multivariate linear regression coefficient estimates with varying lag periods between the 1990 U.S. state-level Gini coefficient and TICS-m score (1998–2010), Health and Retirement Study. (DOCX)

S5 Table Weighted multivariate linear regression coefficient estimates with varying lag periods between the 1990 U.S. MSA-level Gini coefficient and TICS-m score for individuals assessed at every wave (1998–2010), Health and Retirement Study. (DOCX)

S6 Table Weighted multivariate linear regression coefficient estimates with varying lag periods between the 1990 U.S. state-level Gini coefficient and TICS-m score for individuals assessed at every wave (1998–2010), Health and Retirement Study. (DOCX)
