# Supplementary material for: Lagged Associations of Metropolitan Statistical Area- and State-Level Income Inequality with Cognitive Function: The Health and Retirement Study
Source: PLoS One. 2016 Jun 22;11(6):e0157327. doi: 10.1371/journal.pone.0157327 (PMC4917220; doi:10.1371/journal.pone.0157327)
Supplement: S6 Table — (DOCX) [file pone.0157327.s006.docx]

**S6 Table. Weighted multivariate linear regression coefficient estimates with varying lag periods between the 1990 U.S. state-level Gini coefficient and TICS-m score for individuals assessed at every wave (1998-2010), Health and Retirement Study.**

|  | |  | HRS Cohort (n = 4,049 in 1992) | | | | | | | | | | | |  | | |
| --- | --- | --- | --- | --- | --- | --- | --- | --- | --- | --- | --- | --- | --- | --- | --- | --- | --- |
|  | |  | β | |  | | | | 95% CI | | | | | |  | |  |
| 1998 TICS | n=4,049 (100%)  8-year lag |  | |  | | | |  | | | | | |  | |  |  |
|  | Quartile of  1990 Gini |  | |  | | | |  | | | | | |  | |  |  |
|  | Q2 | -0.48* | |  | | -0.95, -0.01 | | | |  | | | |  | |  |  |
|  | Q3 | 0.07 | |  | | -0.33, 0.46 | | | |  | | | |  | |  |  |
|  | Q4 | -0.13 | |  | | -0.59, 0.33 | | | |  | | | |  | |  |  |
|  | | *P for trend = 0.51* | |  | |  | |  | | | | |  | | |  |  |
| 2000  TICS | n=4,049 (100%)  10-year lag |  | |  | | | |  | | | | | |  | |  |  |
|  | Quartile of  1990 Gini |  | |  | | | |  | | | | | |  | |  |  |
|  | Q2 | -0.44 | |  | | -1.00, 0.12 | | | |  | | | |  | |  |  |
|  | Q3 | -0.26 | |  | | -0.78, 0.26 | | | |  | | | |  | |  |  |
|  | Q4 | -0.45 | |  | | -1.05, 0.15 | | | |  | | | |  | |  |  |
|  | | *P for trend = 0.30* | |  | |  | |  | | | | |  | | |  |  |
| 2002  TICS | n=4,049 (100%)  12-year lag |  | |  | | | |  | | | | | |  | |  |  |
|  | Quartile of  1990 Gini |  | |  | | | |  | | | | | |  | |  |  |
|  | Q2 | -0.09 | |  | | -0.53,0.36 | | |  | | | | |  | |  |  |
|  | Q3 | -0.01 | |  | | -0.39,0.38 | | |  | | | | |  | |  |  |
|  | Q4 | 0.00 | |  | | -0.52,0.52 | | |  | | | | |  | |  |  |
|  | | *P for trend = 0.84* | |  | |  | |  | | | | |  | | |  |  |
| 2004  TICS | n=4,049 (100%)  14-year lag |  | |  | | | |  | | | | | |  | |  |  |
|  | Quartile of  1990 Gini |  | |  | | | |  | | | | | |  | |  |  |
|  | Q2 | 0.03 | |  | | -0.34,0.38 | | | |  | | |  |  |  |  |  |
|  | Q3 | 0.09 | |  | | -0.26,0.37 | | | |  | | |  |  |  |  |  |
|  | Q4 | 0.09 | |  | | -0.25,0.44 | | | |  | | |  |  |  |  |  |
|  | | *P for trend = 0.68* | |  | |  | |  | | | | |  | | |  |  |
| 2006  TICS | n=4,049 (100%)  16-year lag |  | |  | | | |  | | | | | |  | |  |  |
|  | Quartile of  1990 Gini |  | |  | | | |  | | | | | |  | |  |  |
|  | Q2 | 0.33 | | |  | | -0.54,0.61 | | | |  | | |  |  |  |  |
|  | Q3 | 0.32 | | |  | | -0.38,0.56 | | | |  | | |  |  |  |  |
|  | Q4 | 0.11 | | |  | | -0.39,0.56 | | | |  | | |  |  |  |  |
|  | | *P for trend = 0.86* | |  | |  | |  | | | | |  | | |  |  |

**S6 Table—Continued.**

|  | |  | | HRS Cohort | | | | | | |  |  |
| --- | --- | --- | --- | --- | --- | --- | --- | --- | --- | --- | --- | --- |
|  | |  | | β | |  | | | 95% CI | |  | |
| 2008  TICS | n=4,049 (100%)  18-year lag | |  | |  | |  | | | |  |  |
|  | Quartile of  1990 Gini | |  | |  | |  | | |  |  |  |
|  | Q2 | | 0.04 | |  | | -0.59,0.66 | | | |  |  |
|  | Q3 | | 0.08 | |  | | -0.28,0.44 | | | |  |  |
|  | Q4 | | -0.01 | |  | | -0.50,0.48 | | | |  |  |
|  | *P for trend = 0.99* | | |  | |  | |  | | | |  |
| 2010  TICS | n=4,049 (100%)  20-year lag | |  | |  | |  | | | |  |  |
|  | Quartile of  1990 Gini | |  | |  | |  | | | |  |  |
|  | Q2 | | 0.11 | |  | | -0.47,0.70 | | | |  |  |
|  | Q3 | | 0.30 | |  | | -0.07,0.67 | | | |  |  |
|  | Q4 | | 0.06 | |  | | -0.33,0.45 | | | |  |  |
|  | *P for trend = 0.65* | | |  | |  | |  | | | |  |

Note. β = coefficient estimate; CI = confidence interval. Q = quartile of 1990 state Gini coefficient.

All models are adjusted for baseline age, gender, race/ethnicity, education, net wealth, self-reported medical diagnoses of diabetes, hypertension, heart disease, and stroke, body mass index, and the 1995/1996 TICS-m score; and the percentage Black and median household income at the state level. Quartile 1 (Q1) or the Gini coefficient (reference category) corresponds to the quartile of US states with the lowest Gini coefficients (lowest level of income inequality), based on all 50 states in 1990. Sample sizes are shown for each outcome year and cohort (the percentage of the baseline sample is shown in parentheses).

* P≤.05.
